# Synergistic NGF/B27 Gradients Position Synapses Heterogeneously in 3D Micropatterned Neural Cultures

**DOI:** 10.1371/journal.pone.0026187

**Published:** 2011-10-13

**Authors:** Anja Kunze, Ana Valero, Dominique Zosso, Philippe Renaud

**Affiliations:** 1 Microsystems Laboratory (LMIS4), Institute of Microengineering, Ecole Polytechnique Fédérale de Lausanne (EPFL), Lausanne, Switzerland; 2 Signal Processing Laboratory (LTS 5), Institute of Electrical Engineering, Ecole Polytechnique Fédérale de Lausanne (EPFL), Lausanne, Switzerland; University of Pennsylvania, United States of America

## Abstract

Native functional brain circuits show different numbers of synapses (synaptic densities) in the cerebral cortex. Until now, different synaptic densities could not be studied *in vitro* using current cell culture methods for primary neurons. Herein, we present a novel microfluidic based cell culture method that combines 3D micropatterning of hydrogel layers with linear chemical gradient formation. Micropatterned hydrogels were used to encapsulate dissociated cortical neurons in laminar cell layers and neurotrophic factors NGF and B27 were added to influence the formation of synapses. Neurotrophic gradients allowed for the positioning of distinguishable synaptic densities throughout a 3D micropatterned neural culture. NGF and B27 gradients were maintained in the microfluidic device for over two weeks without perfusion pumps by utilizing a refilling procedure. Spatial distribution of synapses was examined with a pre-synaptic marker to determine synaptic densities. From our experiments, we observed that (1) cortical neurons responded only to synergistic NGF/B27 gradients, (2) synaptic density increased proportionally to synergistic NGF/B27 gradients; (3) homogeneous distribution of B27 disturbed cortical neurons in sensing NGF gradients and (4) the cell layer position significantly impacted spatial distribution of synapses.

## Introduction

**Figure 1:**
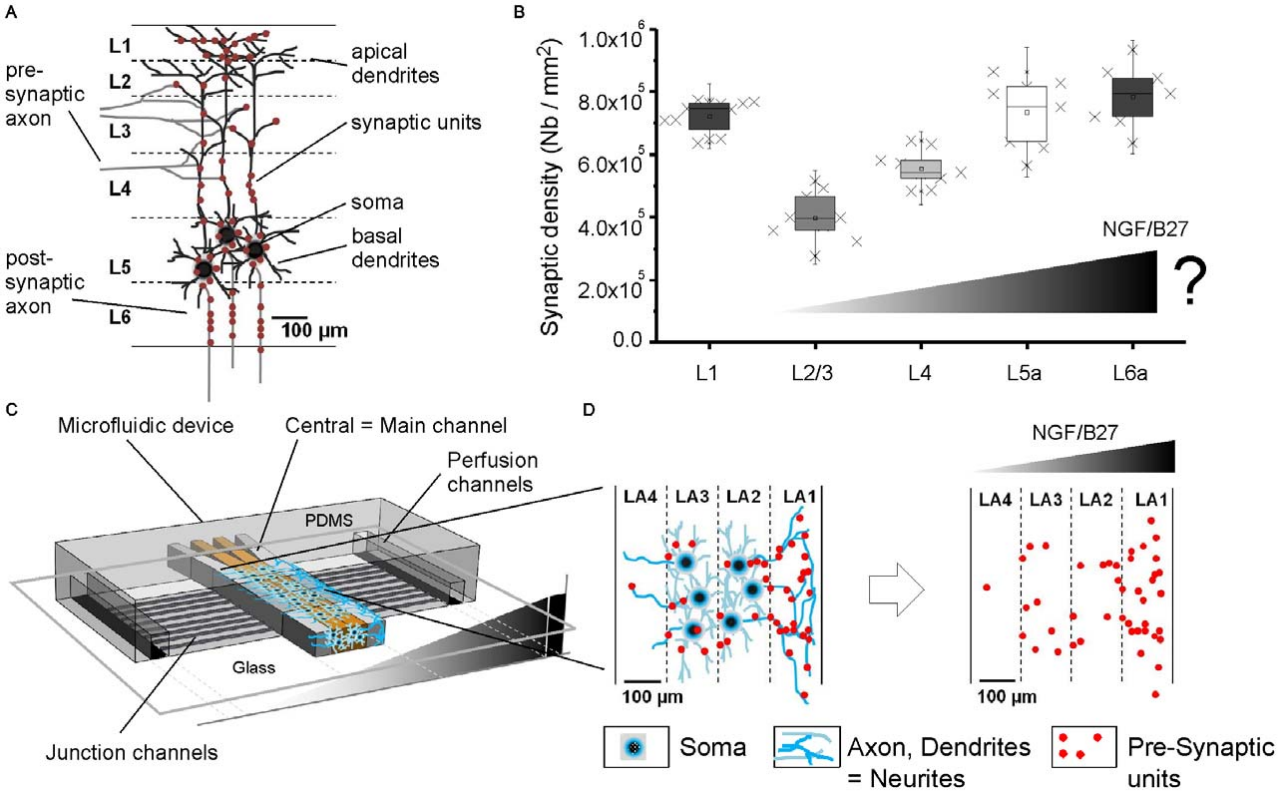
Engineering spatial distribution of synapses in microfabricated 3D neural cell layers. (A) Illustration of native synaptic appearance in the cerebral cortex. Scale adapted to rats. L1 … L6: cortical cell layer notation. (B) Native synaptic density differs within cortical cell layers. Synaptic density was extracted from [3] based on image treatment, described in Supporting information S6. We hypothesize that synergistic NGF/B27 gradients influence axon guidance and spatial distribution of synapses. (C) Schematic view of combining micropatterning and gradient generation in a polydimethylsiloxane (PDMS) microfluidic device. (D) Engineered cortical cell layers and spatial distribution of synapses after B27/NGF gradient exposure. Synergistic gradient guides neurites and increases synapse assembly towards higher concentration. LA 1 … LA 4: hydrogel layer notation *in vitro*.

Engineering the complexity of neurite networks and brain cell architecture *in vitro* is limited by two dimensional neural cell culturing methods. Cortical neurons in their native cell architecture are patterned in six layers (Fig. 1A). An excitatory neural cell consists of a soma, dendrites and an axon. Excitatory neurons are mainly position in the fifth cell layer, L5 where they are surrounded by basal and apical dendrites that spread out toward layer L1 [1]. Axons leave the cerebral cortex through layer L6 by following guidance cues. Incoming axons, from the same or other cerebral regions, bridge to dendrites, soma or axons through synaptic units. A synaptic unit consists of two parts: a pre-synaptic part comprising the incoming axon and a post-synaptic part with the soma or dendrites. Axon to axon connections have also been reported but are rare [2].

Synapses are heterogeneously distributed across all six cortical cell layers (Fig. 1B) and local differences in the number of synapses (synaptic densities) can vary depending on the cell layer [3]. Recent findings suggest a connection between the local appearance of synapses (synapse formation) and neurite guidance factors [4]. Herein, we sought to understand the role of Nerve growth factor (NGF), a known neurite guidance factor that has not yet been directly implicated in influencing synaptic formation.

During brain development, NGF is an important protein for survival and differentiation. Furthermore, NGF repairs nerves [5], [6], guides them in engineered neural tissues [7], [8] and is used in the treatment of Alzheimer's disease [6], [9]. NGF is comprised of three subunits, where β-unit, also known as 2.5S NGF, is the functional portion. The body produces NGF in the peripheral nervous system (PNS), in peripheral tissues, and in the central nervous system (CNS). Neural and non-neural cells in CNS release NGF and engage in paracrine signaling. NGF produced in the PNS is transported through either blood vessels for endocrine signaling or through neurons by retrograde transport mechanisms towards the soma. In the brain, NGF is heterogeneously distributed with higher concentrations in the hippocampus (134±29 ng/ml), than that in the cortex (57±25 ng/ml), cerebellum (42±26 ng/ml) or striatum (16±10 ng/ml) [10]. Since concentration differences generate molecular gradients, we hypothesized that NGF gradients could play a major role in connecting cortical networks and therefore influence synaptic assembly (Fig. 1B).

In case of NGF endocrine signaling, blood vessels close to the white matter and striatum [3], [5] allow for high concentrations of NGF to be released to the cortical cell layer L6. We assumed that NGF is provided in parallel with other trophic factors such as insulin [11]. Insulin is known to affect synapse formation in cell culture [11] and is widely used to enhance neurite outgrowth [12]. However, outgrowth studies of NGF in synergy with insulin have shown conflicting results and seemed to be strongly dependent on cell types [13], [14], [15], [16], [17]. The lack of neurotrophic gradient effects might explain these inconsistent results.

Gradient effects can be studied *in vivo* or *in vitro*. Gradient studies *in vivo* include the microstructured cell architecture, but imposed gradients can interfere with local production and synergistic effects of other trophic factors in the brain [18], [19], [20]. Furthermore, patients often suffer from pain during NGF treatments because high concentrations of NGF are required [5]. Therefore, cell culture methods that mimic 3D connectivity and cell layer architecture *in vitro* are necessary to better understand the influences of brain structure, different synaptic densities, and molecular gradients for brain function.

Cell cultures of dissociated neurons allow for reproducible *in vitro* studies of trophic factors [21], [22]. However, standard culture methods consist of plating cells on two-dimensional surfaces in Petri dishes or multiwells. These culture methods provide only an unstructured, homogeneous environment without defined cell-cell interactions and oriented neurite outgrowth. In the last decade, several groups have used microfluidic devices to improve dissociated cell organization. Local neurite guidance has been achieved with microchannels that connect distinct cell compartments, or that allow for soluble and immobilized concentration gradient patterns [23], [24], [25], [26], [27]. However, these *in vitro* gradient studies containing neural cells are restricted to 2D cell cultures [24], [26], [28], [29], [30]. Gradient studies with 3D neural cell cultures are only available for macro systems with scaffold sizes in the millimeter range (60 mm length x 8 mm diameter), which contradicts the micrometer dimensions of the cell architecture such as found in the cortex, hippocampus, and striatum regions [31], [32].

Here, we present a new microfluidic based culture method that combines a previously published method to pattern neuronal cells in 3D [33] with the ability to establish chemical gradients across the 3D cell layers (Fig. 1C, Supporting information S1). Since synapses are the most important units for neural communication [21], we were interested in engineering spatial synapse distributions based on synergistic NGF and B27 gradients (Fig. 1D). NGF/B27 gradient effects were examined on primary cortical neurons from E19 rats, a cell culture model of the central nervous system (CNS) that is similar to human cell models.

## Results and Discussion

Synergistic NGF and B27 gradient effects were studied on synapse distribution in our micropatterned 3D culture. First, micropatterned neuronal cells were exposed to absolute concentration gradients (∇C) of NGF (∇C_NGF_), B27 (∇C_B27_) or joint NGF/B27 (∇C_NGF_+∇C_B27_). Second, average concentration (C_avg_) of joint NGF/B27 was kept constant and neural cell response was examined on increasing gradients of joint NGF/B27 (↑∇C_NGF_+∇C_B27_, C_avg, NGF_, C_avg, B27_  =  const.). Next, we provided B27 uniformly (C_B27_  =  const.) to the neuronal culture, with increasing NGF gradients (↑∇C_NGF_). The relative concentration gradient (∇C/C_avg_) of NGF was kept constant (Tables 1 and 2). Finally, we also considered changes in cell micropatterning and examined the corresponding synapse formation with respect to the same joint NGF/B27 gradient.

**Table 1:** Concentration gradient formation and neurite outgrowth over two weeks from the artificial neural cell layers (LA 2 & 3) into the adjacent hydrogel layers (LA 1 & 4).

Time	C/C_0_		C_0,NGF_	C_0,B27_	C_0,insulin_	∇NGF	∇B27	∇insulin	ND_left_	SD	ND_right_	SD	ND_rel_
DIV	min	max	ng/ml	%(v/v)	µg/ml	ng/ml/mm	%/mm	ug/ml/mm	N/mm^2^		N/mm^2^		(N_right_-N_left_)/N_left_
2	0.426	0.510	203.87	6.12	12.23	52.5	1.6	3.2	64	12	59	25	-7%
5	0.454	0.538	215.22	6.46	12.91	52.6	1.6	3.2	69	30	69	26	0%
7	0.456	0.540	216.05	6.48	12.96	52.6	1.6	3.2	91	54	145	74	59%
9	0.456	0.540	216.05	6.48	12.96	52.6	1.6	3.2	121	51	154	75	27%

DIV: days *in vitro,* C/C_0_: Relative concentration, C_0, XXX_: Maximal concentration of molecule XXX, C_0,NGF_: 40 ng/ml  =  30.7 µM (13 kDa), C_0,B27_: 12% (v/v), C_0,insulin_: 24 µg/ml  = 4.14 µM (5.8 kDa),∇: gradient, ND: neurite density, SD: standard deviation, n = 5, ND_rel_: Relative neurite difference, N: neurite frequency, right: hydrogel layers LA 1 & LA 2, left: hydrogel layers LA 3 & LA 4.

**Table 2:** Different gradient conditions and their impact on synapse formation and neurite orientation across the main channel.

W_JC_	Experiment	C_0,NGF_	C_0,B27_	C_0,insulin_	∇NGF	∇B27	∇insulin	Slope a	SD a	R	ND_rel_
µm		ng/ml	% (v/v)	µg/ml	ng/ml/mm	%/mm	µg/ml/mm	1/mm	1/mm		(N_right_-N_left_)/N_left_
1000	*0B27pNGF-53*	216.0	0.0	0.0	52.6	0.0	0.0	NA	NA	NA	∼0%
	*2B27*	0.0	2.0	4.0	0.0	0.0	0.0	0.022	0.009	0.02	−8%
	*2B27pNGF-53*	216.0	2.0	4.0	52.6	0.0	0.0	−0.520	0.009	0.58	−12%
	*2B27pNGF-100*	432.1	2.0	4.0	105.1	0.0	0.0	0.106	0.011	0.17	−14%
	*2B27pNGF-200*	864.2	2.0	4.0	210.2	0.0	0.0	0.644	0.010	0.77	−24%
	*12B27*	0.0	6.5	13.0	0.0	1.6	3.2	0.523	0.012	0.65	42%
	*12B27pNGF-53*	216.0	6.5	13.0	52.6	1.6	3.2	0.704	0.003	0.75	27%
150	*12B27pNGF-53*	277.0	8.3	16.6	234.1	7.0	14.0	0.871	0.006	0.93	122%

W_JC_: Junction channel length, C_0, XXX_: Maximal concentration of molecule XXX, ∇: gradient, a: gradient slope factor extracted from linear trend curve fits on synapse puncta in the main channel, SD: standard deviation, n = 5, ND_rel_: Relative neurite difference, N: neurite frequency, NA: non applicable, right: hydrogel layers LA 1 & LA 2, left: hydrogel layers LA 3 & LA 4.

### Periodic reservoir refilling establishes constant gradients after 2 days and prevents contamination

**Figure 2:**
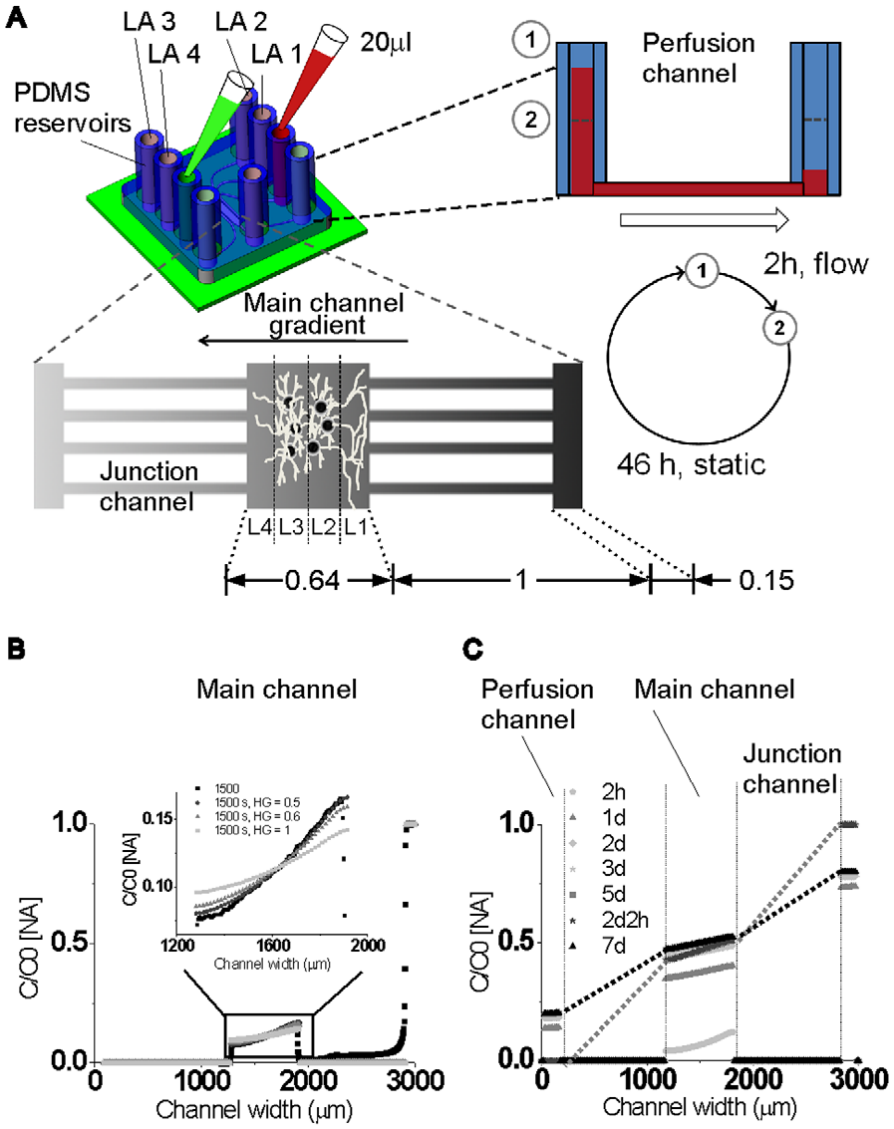
Establishing stable long-term gradients through refilling. (A) Illustration of reservoir refilling procedure. Step 1, empty polydimethylsiloxane (PDMS) reservoirs are selectively filled with medium. Red color indicates enriched NGF/B27 condition. Green color represents pure medium. Stable linear gradients establishes through junction channels and micropatterned hydrogel layers in the main channel, because of perfusion flow. Step 2, after 2 h perfusion flow stops. The long perfusion channels maintain the gradient in the main channel. Dimensions are in mm. Every other day, refilling was repeated. (B) Experimental gradient formation and computational adaption of reduced diffusion in the hydrogel layers in the main channel. (C) Computational stable NGF gradient formation over cell culture period. ∇C stabilizes after 2 h, whereas C_avg_ reaches stable point after 2 d, but before dissociated neurons response.

We developed a periodic reservoir refilling procedure to perform long-term gradient studies in our microfluidic device without perfusion pumps. Figure 2A presents the refilling procedure, which consists of two periodic steps. In the first step, culture medium with desired concentrations of nerve guidance factors is locally injected into incorporated poly(dimethylsiloxane) (PDMS) reservoirs. Volume differences result in pressure driven flow in the perfusion channel. The generation of a soluble gradient is created in the main channel (Fig. 2B, Supporting information S2). During the second step, pressure driven flow reaches hydrostatic equilibrium, and diffusion continues. Reservoirs are refilled every other day. After 2 days, a stable, absolute concentration gradient of ∇C  =  0.131*C_0_/mm is obtained where C_0_  =  C_max_, the maximal added concentration (Fig 2C). Establishing constant chemical gradients earlier than 2 days is not necessary, as immature neurons break of their symmetry in neurite extension after 2 days *in vitro* (DIV) [34], [35]. Using the refilling method, no contamination was observed during the two-week experiment. Hence, our refilling procedure provides reproducible gradient studies without perfusion pumps on micropatterned cell cultures in our microfluidic device.

### NGF-2.5S and B27 gradients act synergistically to form oriented neurite outgrowths

Using the microfluidic device, we micropatterned neural cells embedded in two parallel hydrogel layers in the middle of the main channel surrounded by cell free hydrogel layers. This cell layer formation was chosen to define one single cell layer in the middle of the main channel, facilitating neurite gradient response. Smaller cell layers were avoided to prevent neurite or synapse formation dependent on total covered cell area. Total cell layer width was 234 µm ± 46 µm containing ∼4200 neural cells. Cell-free hydrogel layers were 159 µm ± 41 µm wide on the right side (LA 1) and 176 µm ± 41 µm wide on the left side (LA 4). These micro dimensions of neural cell layers are in consistent with reported literature values of cortical, hippocampal or cerebellar cell layer thicknesses [36], [37], [38].

**Figure 3:**
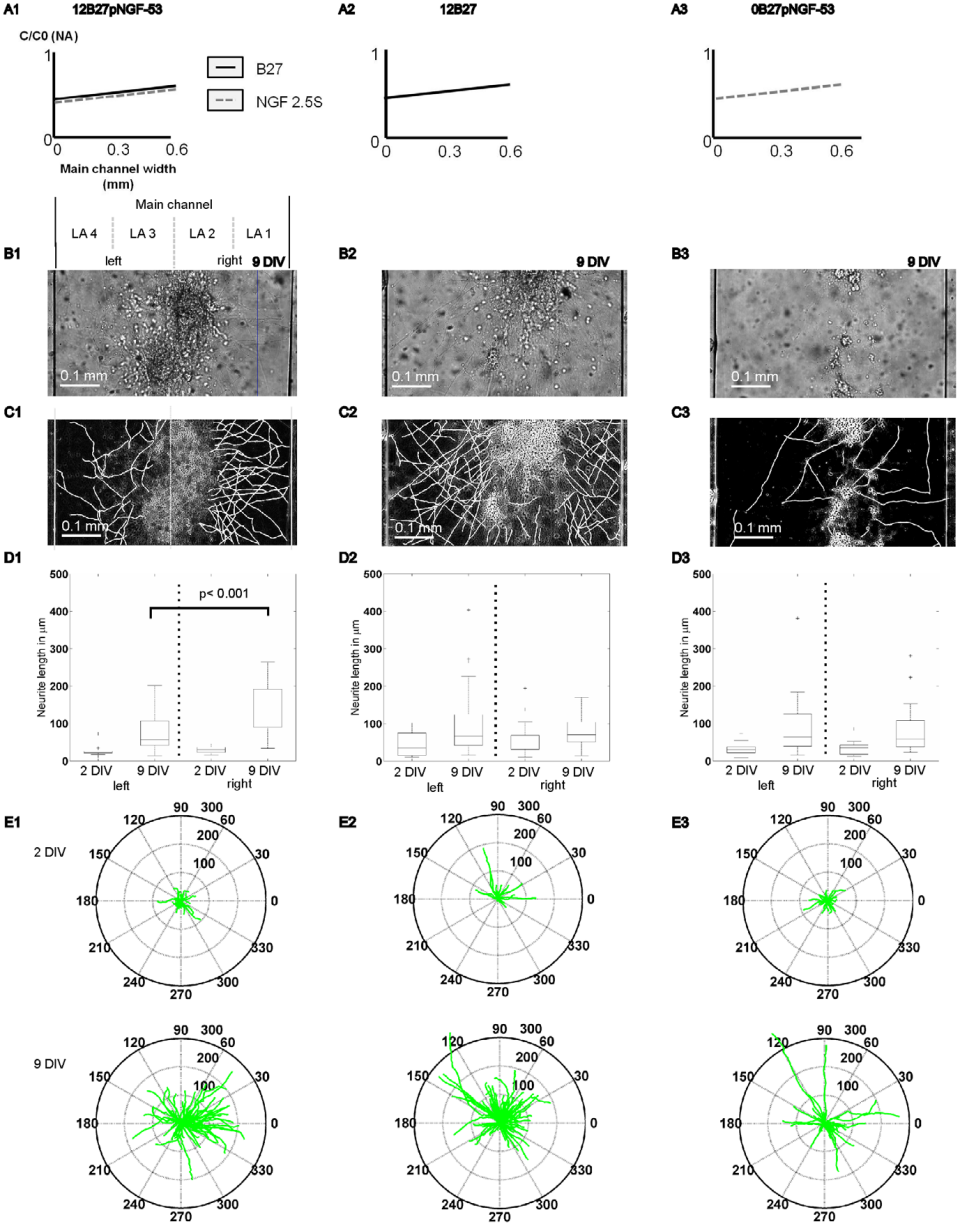
Neurite outgrowth and guidance towards synergistic B27 and NGF gradient. (A, row) Schematic view of single versus synergistic NGF and B27 gradients, which stimulate micropatterned cell cultures in the main channel. (B, row) Differential interference contrast (DIC) images of micropatterned neural cell culture (E19) after 9 days *in vitro* (DIV), bar  = 0.1 mm. (C, row) Inverted DIC images with traced neurites, bar  = 0.1 mm. (D, row) Neurite lengths grown in left versus right hydrogel layers. (E, row) Neurite traces from 2 and 9 DIV, summarized in polar plots. Lengths of radii are in µm and angles are in degree. Only synergistic NGF/B27 orient neurite outgrowth towards higher concentrations.

To study neurite guidance effects of joint ∇C_NGF_+∇C_B27_ (NGF/B27) versus single ∇C_NGF_ or ∇C_B27_ of neurotrophic factors, gradients were generated through the artificial layer LA 1 to LA 4 perpendicular to micropatterned hydrogel layers (Fig. 3A 1–3). Absolute concentrations and gradient values increase from LA 4 to LA 1 (Table 1 and 2).

Previously, neurite outgrowth was reported only for higher NGF gradients ∇C_NGF_ >200 ng/ml/mm for dorsal root ganglia embedded in hydrogel [32], 133 ng/ml/mm –200 ng/ml/mm NGF for pheochromocytoma (PC-12) cell line covered with hydrogel [39], or 833 ng/ml/mm netrin-1 [31]. In contrast, when neurons were subjected to ∇C_NGF_ 53 ng/ml/mm, we observed sparse neurite outgrowth, although there was no preferred orientation or local increased neurite density (Fig. 3, 3^rd^ column, Table 2).

As expected, we observed neurite outgrowth with the joint NGF/B27 gradients (∇C_NGF_: 53 ng/ml/mm, ∇C_B27_: 1.56%(v/v)/mm) towards higher NGF/B27 concentrations in layer LA 1 (Fig. 3, 1^st^ column). Although neurons can respond already after 2 DIV on environmental cues, we found that differences in neurite density, orientation and length were negligible between right (LA 1 + LA 2) and the left (LA 3 + LA 4) hydrogel layers after 2 DIV (Table 1). After 5 DIV, neurons spread out and formed neurites towards higher NGF/B27 concentrations (Table 1). The joint NGF/B27 gradient had the highest impact on neurite outgrowth after 7 DIV, with significant longer neurites towards layer LA 1 (Fig. 3 1D, one-way ANOVA, p<0.001). Interestingly, many neurites oriented towards the steepest concentration gradient (angle range between 60° and −30°, Fig. 3 E1). However, neurite density was not significantly influenced by the NGF/B27 gradient (Table 1).

Jones *et al* showed synergistic effects of absolute concentrations of 25 ng/ml NGF plus 25 ng/ml (IGF-1) that enhanced neurite outgrowth of dorsal root ganglia [16]. Our results also indicate that synergistic gradient effects between insulin and NGF could affect primary cortical neurons.

When NGF was omitted, and micropatterned neurons were exposed to ∇C_B27_ (1.56% (v/v)/mm), differences in neurite length were not significant after 9 DIV between LA 1 and LA 2 (Fig. 3, 2^nd^ column). However, neurite orientation was detected after 2 DIV towards the steepest concentration gradient in layer LA 1 (Fig. 3 E2). This neurite orientation remained parallel to ∇C_B27_ after 9 DIV, even though the effect of different neurite lengths between LA 1 and LA 4 disappeared (Fig. 3 D2). Neurite density showed 42% higher values in LA 1 than in LA 4 under ∇C_B27_ (Table 2). Thus, the single B27 gradient has only an initial guidance effect but a strong effect on neurite local neurite density.

In summary, single NGF gradients without B27 resulted in sparse neurite outgrowth (Fig. 3, 3^rd^ column). In contrast, joint ∇C_NGF_+∇C_B27_ induced oriented neurite outgrowth (Fig. 3, 1^st^ column) with a 60% lower NGF gradient (53 ng/ml/mm) than previously reported gradient values [28], [32].

### Synapse distribution increases with synergistic NGF/B27 gradients

After neurite network formation, synaptic units are an indicator for neural communication [21]. To examine synaptic units, pre-synaptic proteins were stained with synaptophysin. To prove coherent location of pre-synaptic units on axons, neurons were stained with neurofilament marker NF-L for axons and MAP-2 for dendrites (Supporting information S3). Synaptophysin puncta follow NF-L stained axons, whereas MAP-2 co-localized with only a few synaptophysin puncta, providing evidence of the existence of synaptic units between axons and dendrites (Supporting information S3).

**Figure 4:**
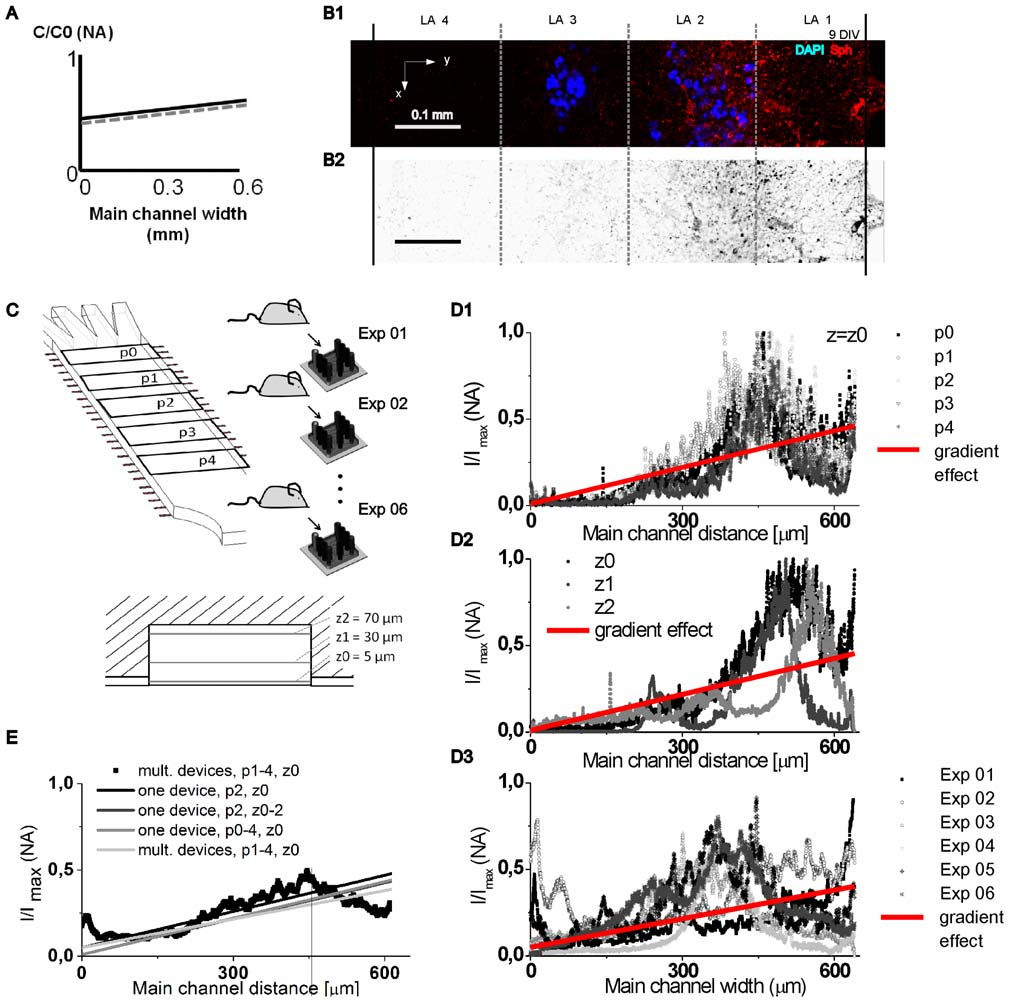
Using synergistic NGF/B27 gradients polarizes spatial synapse distribution towards higher concentrations. (A) Schematic view of synergistic NGF/B27 gradients in the main channel. (B1) False color images shows micropatterned cell layers through nuclei staining (DAPI, blue) and polarized pre-synaptic units (Synaptophysin, red). (B2) Inverted red channel highlight synapse distribution. (C) Evaluation parameters. (D, column) Surface plot of spatial synapse distribution and linear regression fit of data. D1: different lateral positions, D2: different vertical positions and D3: different experimental batches. (E) Averaged spatial synapse distribution correlates with linear fit of data (gradient effect) and is independent of evaluation parameters.

We analyzed synapse formations dependent on joint NGF/B27 gradients (∇C_NGF_: 53 ng/ml/mm, ∇C_B27_: 1.56% (v/v)/mm) with two different methods, including the use of spatial surface intensity plots (Fig. 4, Supporting information S4) and the determinations of local synaptic densities (Supporting Information S6). Normalized intensity plots (I/I_max_) of synaptophysin fluorescence signal show an increased accumulation of synapses in LA 2 and LA 1 in selected regions of interest (Fig. 4 B1, B2, D1–3, ROIs, 640 µm x 200 µm) at multiple selected positions in the main channel (Fig. 4 C). Synaptic units increased in correlation to the steepest portion of joint NGF/B27 gradients. Spatial distribution of synaptophysin puncta was independent of selected lateral and z-positions in the main channel. Averaging synaptophysin puncta distribution over multiple experiments (n = 6) in different microfluidic devices, with different origins of neural cells, revealed linear correlations between increasing synaptic densities and joint NGF/B27 gradients (Fig. 4 E).

Synaptic density quantification revealed 40% more synaptophysin puncta in the artificial hydrogel layer LA 1 as compared to layer LA 4 (one-way ANOVA, p<0.001, n = 16) after 9 DIV and joint NGF/B27 gradient exposure and different puncta in LA 1 and LA 2 (Supporting information S3).

To show that synapse distribution follows increasing absolute ∇C and relative ∇C/C_avg_ NGF/B27 gradients across the main channel, we performed cell culture experiments in a microfluidic device with shorter junction channel lengths. Longer junction channels (L = 1 mm) were initially designed to maintain chemical gradients as long as possible by increasing the diffusive length during the second step of the refilling method. The longer the diffusive length, the lower ∇C and ∇C/C_avg_ will be across the hydrogel. Decreasing the length of junction channels by a factor 6.7 (final length L  = 0.150 mm) increased the absolute joint NGF/B27 gradient by 28.0%±2.5 (Supporting information S5). After 9 DIV, we compared synapse formation in the micropatterned neural culture over the main channel width. An increased synaptic density of 23.7%±0.6 was seen in the short compared to the long junction channel device (Table 2). The linear increase of synaptic density was slightly lower than the increase of the gradient slope ∇C. It seems that synaptic density saturates at high NGF/B27 concentrations. This saturation effect may be due to a faster decrease of the gradient during Step 2 of the refilling method or a limitation in cellular function. However, synapse formation follows increasing joint NGF/B27 gradients in our micropatterned neural cell culture.

### Homogeneous distribution of B27 disrupts synapse formation in NGF gradients

**Figure 5:**
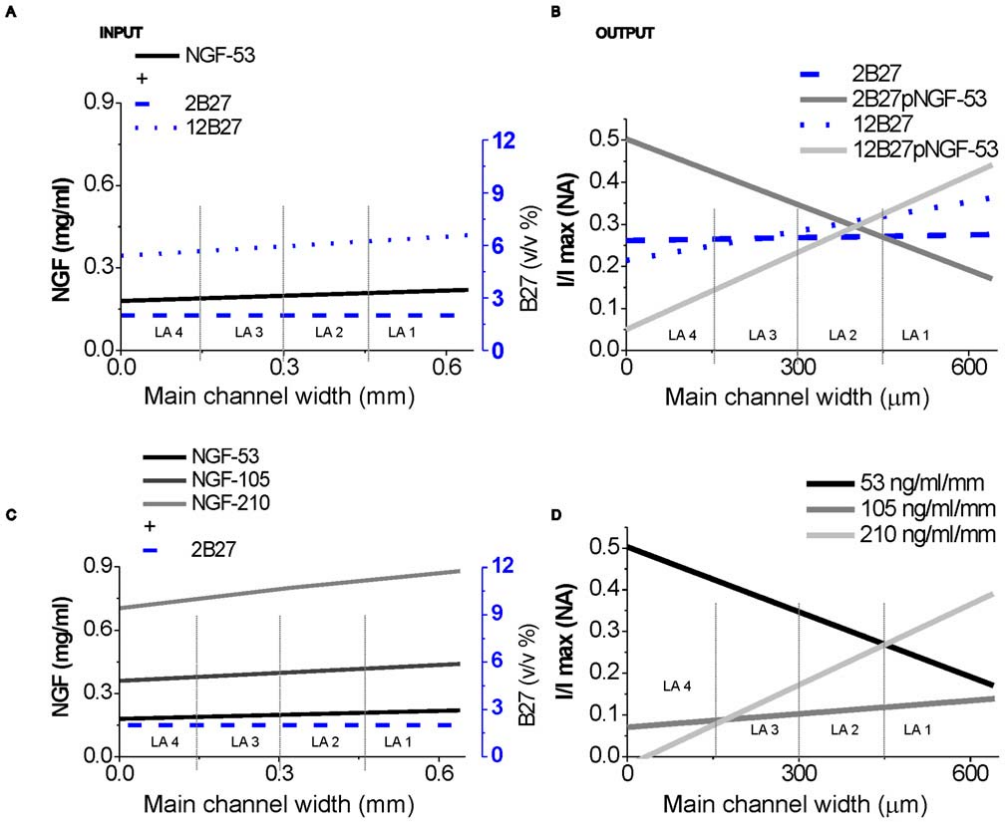
Different combinations of synergistic NGF/B27 gradients impact spatial synapse distribution. (A and C) Gradient input in the main channel. (A) Stable NGF gradient was combined with a homogenous B27 distribution or a B27 gradient. (B and D) Linear regression fits from synapse distribution. (B) Homogenous B27 distribution disturbs NGF gradient sensing. Cortical neurons express polarized synapse distribution only when synergistic NGF/B27 gradients interplay. (C) Homogenous B27 distribution was combined with increased ∇C/C_avg_ NGF gradients. (D) Disturbed synapse distribution was recovered through high relative NGF gradients.

Neural cells are often cultured with Neurobasal and uniform concentrations of B27 supplement [12]. However, the absence of a B27 gradient resulted in sparse neurite outgrowth without orientation when NGF gradients were still present. This raised the question whether a uniform distribution of B27, in conjunction with a NGF gradient, would yield the same synapse distribution compared to a joint NGF/B27 gradient. Micropatterned neural cells were exposed to two further concentration profiles based on a homogeneous concentration of 2% (v/v) B27 (Fig. 5A). Synapse distribution was evaluated after providing B27 supplement in uniform or gradient conditions with joint ∇C_NGF_ (53 ng/ml/mm).

3D micropatterned neural cells cultured under uniform B27 conditions (2B27) presented homogeneous neurite outgrowth (Table 2) and spatial synapse distribution in left and right hydrogel layers (Fig. 5B). These results are similar to the neurite outgrowth effects seen from B27 supplements [40], [41], but without the presence of guidance or orientation effects. However, our observations of neurite density and synapse distribution correlated with increased B27 concentrations in layer LA 1 (Table 2).

A homogeneous distribution of B27 in synergy with the NGF gradient (2B27pNGF-53) oriented axons after 9 DIV towards the artificial layer LA 4 with increasing or decreasing neurite density ratios (Table 2). Neurite orientation is reduced at steeper parts of the NGF gradient, and synapse formation followed the new orientation of axons. Therefore, synaptic density decreased from the right artificial layer LA 1 to the left layer LA 4 (Table 2).

A 3.1% increase in the average concentration of B27 ingredients (which is composed primarily of insulin) in layer LA 1 guided neurites and synapse formation towards higher NGF concentration gradients (Fig. 5, 12B27pNGF-53 condition). We observed that a homogeneous concentration of insulin (2B27pNGF-53 condition) triggered sensing of low NGF concentrations (C_avg_ of NGF in layer LA 4: 190 ng/ml). These conditions may explain the conflicting results with NGF and insulin seen previously in neurite formation studies. Furthermore, misaligned spatial synapse distribution can be interpreted as a disruption of NGF gradient sensing, which probably occurs in mental disorders where insulin is involved.

The effect on synapse distribution when B27 is supplied homogeneously or in a gradient together with NGF confirms: (1) the capability of ∇C_B27_ to generate local differences in synaptic densities; (2) the existence of synergistic effects between joint NGF/B27 gradients and (3) the occurrence of misaligned synapse formation, which leads to dysfunction of the neural network, when B27 was distributed homogeneously.

Since NGF is known to rearrange misaligned neurite networks [6] and to restore network functions [9], we sought to evaluate an absolute ∇C_NGF_, while keeping the relative gradient ∇C_NGF_/C_avg_ constant (Fig. 5C and Table 2, 2B27pNGF-100, -200). Again, when comparing synapse distributions tendencies, higher synaptic density were seen for neurons in layer LA 1 with increasing NGF concentration (Fig. 5D). The relative difference of neurite density is reported as negative, which means that neurite density in LA 4 is higher than in LA 1 (Table 2). Hence, increasing ∇C_NGF_ did not reorient misaligned neurites towards higher NGF concentrations, but did restore neurite network function; thus, increasing synaptic density in LA 1 correlated with increasing ∇C_NGF_.

### Cell layer micropatterning influences synaptic density

**Figure 6:**
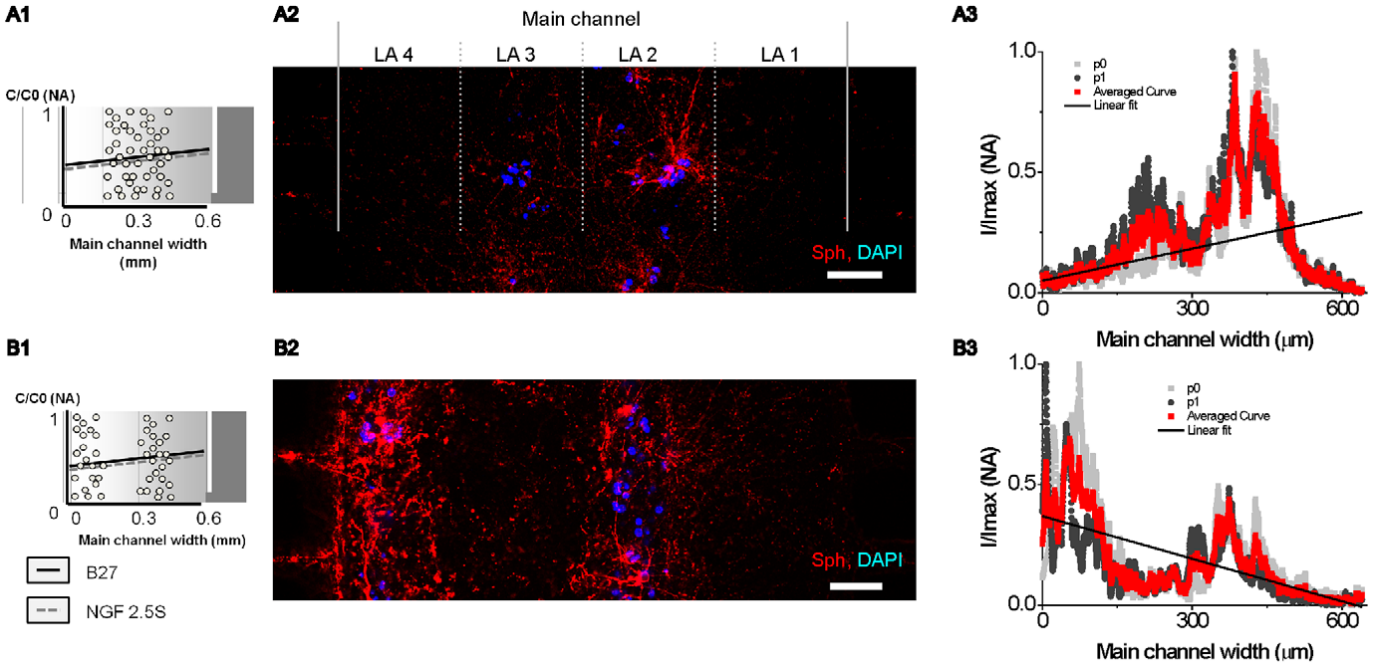
Cell layer position influences spatial synapse distribution. (A1 and B1) Schematic view of shifted cell layer position and NGF/B27 (12B27pNGF-53) gradient exposure. (A2 and B2) False color image shows cell pattern dependent synapse distribution in the micropatterned cell culture after 9 DIV. Pre-synaptic units: synaptophysin (red), cell nucleus: DAPI (blue), scale bar  = 100 µm. (A3 and B3) Surface plots of synapse distribution with linear fit of data demonstrate synaptic gradient response, z  = 5 µm.

Cells are heterogeneously distributed in the brain, and this distribution changes within 100 µm to 200 µm. Therefore, cell layer positioning might influence neural cell responses. To study the influence of a modified cell patterning on synapse formation under the joint B27-NGF gradient, cells from layer LA 3 were shifted to layer LA 4 (Fig. 6 A1 and B1). Total cell density and NGF/B27 gradients (53 ng/ml/mm – 1.6%/mm) remained constant. False color images show increased synaptic density around cortical neurons in layer LA 4 compared to their position in LA 3 (Fig. 6 A2 and B2). In addition, local NGF/B27 concentrations are higher in LA 3 than in LA 4 because of the gradient. We observed axons in layer LA 3, which connect neurons between LA 4 and LA 2, but also axons in LA 1, oriented towards higher NGF/B27 concentrations. Beside spatial synapse distribution (Fig. 6 A3 and B3), local synaptic density was determined in regions of interests (ROIs) of 50×50 µm^2^ layer-by-layer (Supporting information S6).

Comparing synaptic density in separated and non-separated cell layers resulted in no significantly different means between both layers LA 1 and LA 2 (ANOVA, two way, p<0.005, n = 16 ROIs). The average number of synaptic densities in layers LA 1 and LA 2 was 70.2±47.5×10^3^ synapses/mm^2^ and 413.4±165.0×10^3^ synapses/mm^2^, respectively. Neurite outgrowth and synaptic density in layer LA 1 and LA 2 were independent of the changed cell pattern. We can also conclude that cells located more than 320 µm away from structural changes were insensitive to these changes, which is in accordance to literature, where neural cells responded to a laminin pattern up to 100 µm away [42].

Synaptic densities in LA 3 and LA 4 are significantly different (ANOVA, two way, p<0.001, n = 16 ROIs). Cells that were shifted from LA 3 to layer LA 4 were exposed to a lower concentration of NGF/B27, but they generated ∼100% more synapses in LA 4 than that in layer LA 3. The addition of cells in layer LA 4 resulted in ∼400% more synapse per mm^2^ as compared to the cell free condition. We assume that separated cell layers generated an additional NGF gradient and low concentrations of insulin in layer LA 4 probably triggered a different NGF sensing pathway at low NGF concentrations. NGF can be released by cortical pyramidal cells and act as a paracrine factor on neural and non-neural cells [5]. We hypothesize those pyramidal cells from layer LA 2 produce NGF that added a second ∇C_NGF_ that boosted synapse formation in LA 4.

In summary, the location of cells in the micropatterned neural cell culture significantly changed neural cell response, which opens promising new further studies in neuroscience to understand the influence of cortical thickness heterogeneity on cortical brain function under cortical atrophies [43], Alzheimer's disease [44] or schizophrenia [45].

### Conclusion

Here, we demonstrated an enhanced microfluidic based 3D neural cell culture method that allows studying interactions between molecular gradients and cell layer architecture. We found that: (1) Structure and length of hydrogel layers represent natural micro dimensions, as they can be found in the cortex, hippocampus or cerebellum cell layers. Multiple chemical gradients were applied to the micropatterned neural culture to study oriented neurite outgrowth and spatial synapse formation. (2) Establishing engineered chemical gradients with our proposed refilling procedure enables long-term cell culture gradient studies over two weeks with minimized risk of contaminations. (3) We demonstrated the capability of synergistic NGF/B27 (∇C_NGF_ + ∇C_B27_) gradients to polarize spatial synapse formation across 3D micropatterned neural cell cultures. Also, ∇C_B27_ generate local differences in synaptic density. Furthermore, homogenous distribution of B27 disturbed NGF gradient sensing of cortical neurons, which can be restored by increasing ∇C_NGF_. Finally, (4) modifying local cell position in the micropatterned neural culture significantly changed spatial synapse distribution, which opens promising new further studies in neuroscience to understand the influence of cortical thickness heterogeneity. Our novel experimental technique provides new control mechanisms on engineering the neural microenvironment *in vitro* with combined molecular gradient formation. Such a novel system facilitates enriching our knowledge about cell layer interaction with molecular pathway mechanism in neurodegenerative diseases, schizophrenia or mental disorders.

## Materials and Methods

### Ethics Statement

Primary cortical neurons were obtained from embryonic rats (Wistar, E19) in accordance to all state and federal regulations of the Canton Vaud in Switzerland (Approved by Réseau des animaleries lémaniques, Licence No. 1853.1). Pregnant female rat and embryonic rats were anesthetized before decapitation to prevent any pain. The Réseau des animaleries lémaniques (RESAL) is the cantonal veterinary authority of the canton Vaud in Switzerland that approves animal experiments also based on an ethical evaluation. A separate local institutional ethical committee is not involved, because our specific kind of experiments comprises only the culture of the extracted cells, which is considered as non-sentient material.

### Materials

Nerve growth factor (NGF-2.5S, murine gland), Agarose powder type VII, poly(ethylenimine) (PEI), fluorescein sodium salt, 10x PBS pH 7.4, bovine serum albumin (BSA), Tris/HCl, 4′,6-Diamidino-2-phenyindole (DAPI, dilactate) and formaldehyde were purchased from Sigma-Aldrich. Alginic acid was obtained from Medipol. Neurobasal medium, horse serum and B27 were acquired from Invitrogen. The microfluidic chip consists of poly(dimethylsiloxane) (PDMS Sylgard 184) purchased from Dow Corning. The syringe pump (neMESYS Basis-Module 12) was acquired from centoni GmbH and the glass syringes from Ils microsyringes. Formaldehyd and Triton-X-100 were purchased from Merck. Primary antibodies: NF-L (rabbit), MAP-2 (rabbit) and Synaptophysin (mouse) were provided by LubioScience. Secondary antibodies goat-anti rabbit IgG (Cy-2 conjugated) and goat-anti mouse IgG (rhodamine conjugated) were purchased from Dianova.

### Microfluidic device fabrication for neural cell cultures

The microfluidic design is an extended version of the previously published four layer design[33]. It consisted of three parts: the main channel, two parallel perfusion channels and 2×24 junction channels, see supporting information S1. Additional novel features are the long junction and perfusion channels. Junction channels provided nutrient supply from the perfusion channels to the main channel and were designed to maintain a chemical gradient over 3 days. Furthermore, they maintained laminar formation of 3D hydrogel and facilitated stable channel wall fabrication in PDMS. The 24 junction channels were 1 mm long, 10 µm high and 20 µm wide and connect the perfusion channels with the main channel. The two perfusion channels were in total 20 mm long, 0.15 mm wide and 0.1 mm high.

Microfluidic device fabrication consisted of two steps: (1) silicon master fabrication using 2-step dry etching process, previously described [46] and (2) poly(methylmethaacrylate) injection molding previously described [33]. In literature, two different structure heights of the master mold have been achieved by a two-step photolithography with SU-8 [24], [25], [47]. We chose a two step silicon dry etching process, because of the longer life span of silicon structures compared to photoresist structures during PDMS molding (Supporting information S1). PDMS microfluidic devices were assembled on 18 mm x 18 mm glass slides using oxygen plasma bonding (50 W, 0. sccr, 0.3 mtorr, 45 s).

### Micropatterning of primary cortical neurons in hydrogel layers in microfluidic devices

The protocol has been previously described in [33]. In brief: cortical tissues were extracted and washed in PBS + 33 mM glucose and subsequently digested for 15 min at 37°C in 1% (v/v) papain (25 mg/ml) in Segal's medium adjusted to pH 7. Supernatant with neural cells was transferred to Neurobasal/horse serum (10% (v/v)) and filtered. 120×106 cells/ml were mixed (1∶1 v/v) with a hydrogel mixture containing 0.5% (w/v) agarose and 0.3% (w/v) alginate and kept at 37°C until injection in the microfluidic device.

For the micropatterning of the hydrogel layers in the main channel, all channels were filled with Neurobasal/Penstrep (1% v/v). The outlet of the microfluidic device was connected to a syringe pump. Inlets 2 and 3 of the main channel were filled with 20 µl of cell-hydrogel solutions and inlets 1 and 4 with 20 µl medium hydrogel solution. The hydrogel layer formation follows a previously described protocol [33]. After gelling of the hydrogel layers, the devices were placed in the incubator until B27 and NGF enriched medium was injected for neurite stimulation.

### Chemical gradient generation

To generate a linear chemical gradient through the 3D microenvironment in the main channel, the concentrations in the perfusion channels have to be kept constant. This is guaranteed when the perfusion channels are perfused with a molecular flow rate Q_mol_ about twice the diffusive flow Q_diff_. The diffusive flow is the product of the diffusive flux *j* through a surface *A* (eq. 1). Applied to our microfluidic design the diffusive flow can be calculated by equation 2.
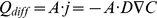
(1)

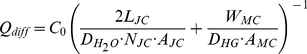
(2)


The flow rate depends upon the diffusion coefficient D of the molecule in the hydrogel (D_HG_) and in medium (D_H20_), upon maximal injected concentration C_0_, upon the number N_JC_ of junction channels (JC), their length L_JC_ and cross section A_JC_, the width W_MC_ and cross section A_MC_ of the main channel. The molecular flow rate Q_mol_ is equal to the convective flow in the perfusion channels Q_conv_ multiplied by the maximal injected concentration. It can be calculated by equation 3:
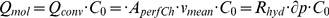
(3)


Here, A_perfCh_ is the cross section and v_mean_ the average flow velocity in the perfusion channel.

We designed our gradient generation procedure based on a refilling method of the PDMS reservoirs. First, both PDMS reservoirs of the perfusion channels are emptied. Second, 20 µl were injected in one of the PDMS reservoir. The volume difference between two connected reservoirs generates a flow until equilibrium (δ*p* = 0) is reached after 2 h. During the 2 h, a chemical gradient ∇C establishes. After 2 h, molecules diffuse from the higher to the lower concentrated perfusion channels and out into the medium in the reservoirs. Every other day the medium in the reservoir is changed.

### Chemical gradient visualization and simulation

After hydrogel formation in the main channel, 20 nM fluorescein and PBS were injected using the refilling method, fluorescein in one reservoir of the right perfusion channel and PBS in one of the left. To analyse the fluorescein gradient during perfusion, fluorescence images were acquired with a Leica microscope every 5 min (Hg lamp, FITC filter). Intensity plots of the gradient in the main channel were normalized with a fluorescence plot from a device completely filled with fluorescein. The intensity plots were transformed into concentration plots by a previously established concentration characteristic. Concentration plots, achieved by 2D COMSOL simulation solving the diffusion equation for the boundary conditions: concentration in the left perfusion channel C_left_  = 0 and in the right perfusion channel C_right_  = 1 at different time steps, were compared to experimentally achieved concentration plots. Matching experimentally achieved concentration gradients in the main channel with the simulated one, the diffusion coefficient D_HG_ in hydrogel could be derived from the diffusion coefficient D_H2O_ in water according to equation 4: 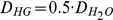
(4)


Our correlations are based on a diffusion coefficient for fluorescein (f) D_f_  =  from 540 µm^2^/s taken from literature [48]. NGF is a molecule consisting of 13 kDa polypeptide chains with a diffusion coefficient in the brain ∼27.5 µm2/s [49]. Using the diffusion coefficient of NGF in PBS instead of water, D_NGF_  = 126 µm^2^/s [49], we estimated the different gradient parameters during the gradient study.

### Different multiple gradient conditions

A linear chemical gradient can be characterized by its absolute concentration gradient ∇C (eq. 5), average concentration C_avg_ (eq. 6) and relative concentration gradient ∇C/C_avg._

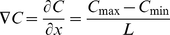
(5)

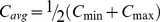
(6)


To study synergistic gradient effects between NGF and B27, which contains insulin, four different experimental conditions have been designed. These conditions can be distinguished based on uniform distribution (∇_B27_  = 0, C_avg,B27_  = 2% (v/v)), gradient distribution (∇C_B27_) and omitted B27 concentrations (C_avg,B27_  = 0). Additionally, NGF gradients (∇C_NGF_) were added. This gives the following five combinations of NGF and B27 culturing conditions: (1) 12B27pNGF-53 = 12% (v/v) B27 plus 53 ng/ml/mm NGF (joint NGF/B27 gradient); (2) 12B27 =  12% (v/v) (B27 gradient); (3) 2B27pNGF-53 = 2% (v/v) B27 (no gradient) plus 53 ng/ml/mm (NGF gradient with B27 constant); (4) 2B27 = 2% (v/v) B27 (no gradient) and (5) 0B27pNGF-53 = 53 ng/ml/mm (pure NGF gradient). In two additional experiments we (1) increased ∇C/C_avg_ keeping C_avg_ constant for the joint NGF/B27 gradient and (2) increased C_avg_ of NGF, keeping ∇C/C_avg_ of NGF and ∇C_B27_  = 0, C_avg,B27_ = 2% (v/v)constant.

### Immunocytochemistry

Neural cells in the micropatterned hydrogel layers were fixated with 4% (v/v) formaldehyde in PBS, injected into all microchannels and left for 25 min. Then reservoirs were emptied. Microfluidic channels were re-filled with 3% (v/v) BSA in 0.1% (v/v) triton/PBS and incubated for 45 min before primary antibody treatment. Primary antibodies (synaptophysin, NF-L, 1∶150 in PBST) were injected into opposite reservoirs and incubated for 2 h. Microfluidic channels were washed three times with PBST and filled in dark with the CY-2 or rhodamine coupled secondary antibody (1∶150 in PBST) and incubated over night at room temperature. After washing with PBS (3x), cell nuclei were stained with DAPI (1∶7000 in PBS) for 20 min and washed with PBS (3x).

### Image acquisition and data analysis for neural cell culture

Neurite outgrowth was examined under a differential interference contrast (DIC) microscope (Zeiss Axiovert 200, digital camera AxioCam HSc) every other day in regions of interest (ROI, 0.64 mm width, 0.2 mm length). For quantitative analyses of neurite lengths and orientation, a previously described image processing [33] was performed with ImageJ to enhance neurite contours. In addition, neurites were traced with NeuronJ and their frequency, lengths and vertexes were extracted to determine the length of neurite outgrowth, neurite density per mm^2^ and orientation. Box-plots, polar plots and analysis of variance (ANOVA) were performed with MATLAB.

After fixation and immunostaining, micropatterned neural cultures were observed under confocal microscopy (Zeiss LSM 700 inverted). For synaptophysin detection, a solid state laser was used at a wavelength of 555 nm with emission filter BP 575–640. DAPI was excited with a Diode laser at 405 nm and detected with emission filter BP 420–470. NF-L and MAP-2 staining have been coupled to CY-2 that was excited by an argon laser at 488 nm and imaged through an emission filter BP 515–565.

To visualize synapse formation and differences in density evoked through the gradient supply of NGF and B27 supplement, averaged fluorescence intensity in ROIs (0.64 mm width, 0.2 mm length) were surface plotted over the main channel width. Fluorescence intensity surface plots were normalized to the maximum and minimum intensity. The increase of synaptic density was examined by linear regression fits using equation 7 on multiple plots (5 different positions in the main channel, 3 different z-positions, 3 to 6 different devices within the same experimental condition). As neural cells, exposed to uniform concentrations, expressed uniform neurite outgrowth and synaptic density, we assumed that neuronal cells, exposed to chemical gradients ∇C, will show linear effects in their response. For a generic model of a neural cell culture in our microfluidic device, we assumed uniform distribution of synapses along their axons (Supporting information S5). Plotting synapse frequency derived from uniform culture conditions over the main channel results in a bell-shaped distribution of synapses. Assuming that axons were oriented through a gradient ∇C in the direction of the artificial layer LA 1, without any change of synaptic density distribution per axons and soma, synapse formation should increase linear to ∇C. The synaptic linear trend under gradient culture condition can be visualized through a data point fit to equation 7:
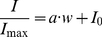
(7)


Here, I_0_ is the minimal and I_max_ the maximal detected fluorescence signal, w is the variable of the main channel position and *a* the slope of the linear regression fit. The slope *a* and I_0_ were fitted for the different experimental conditions and were used to compare synapse formation across the main channel in the different experimental conditions.

## Supporting Information

Supporting Information S1 **Design and fabrication of the microfluidic based cell culture device.** This file gives further details on the microfluidic design and its fabrication steps.
(DOC)

Supporting Information S2 **Chemical gradient characterization and modeling.** Details on gradient measurements and modelling are presented.
(DOC)

Supporting Information S3 **Morphological evaluation through immunostaining.** Chosen immunostainings are explained in detail and non specific binding issues in the hydrogel are discussed.
(DOC)

Supporting Information S4 **Evaluation of the cell response on the NGF/B27 gradient based on synapse formation.** This file gives details how spatial synapse distribution was evaluated based on spatial fluorescence intensity measurements.
(DOC)

Supporting Information S5 **Synapse distribution increases with a higher gradient slope.** Additional results that show the increased spatial synapse distribution through increased gradient slope.
(DOC)

Supporting Information S6 **Evaluation of the cell response on the NGF/B27 gradient based on synaptic density.** This file gives further details on evaluating spatial synapse distribution through determining local synaptic densities.
(DOC)
