# Supplementary material for: Synergistic NGF/B27 Gradients Position Synapses Heterogeneously in 3D Micropatterned Neural Cultures
Source: PLoS One. 2011 Oct 13;6(10):e26187. doi: 10.1371/journal.pone.0026187 (PMC3192785; doi:10.1371/journal.pone.0026187)
Supplement: Supporting Information S1 — Design and fabrication of the microfluidic based cell culture device. This file gives further details on the microfluidic design and its fabrication steps. (DOC) [file pone.0026187.s001.doc]

*Design and fabrication of the microfluidic based cell culture device*

Briefly summarized: first, 1 µm silicon wet oxide was grown on a silicon wafer at 1050°C. The silicon oxide functioned as a mask for the second dry etch step, achieving the final channel height of 100 µm, and was structured by standard photolithography. A 1 µm thick layer of AZ 1518 photoresist was spun on the oxide and exposed to UV light through a chrome mask. The 2nd photolithography step structured the junction channels. A 5 µm thick layer of AZ 9260 photoresist was coated on the structured oxide layer. The resist height had to be five times higher to cover as planar as possible the 1 µm thick oxide structure. Silicon junction channels were 10 µm etched into the planar silicon surface using deep reactive ion etching (DRIE). The photoresist layer was removed and the final microfluidic channel structure etched using the BOSCH process. A final wet-oxidation process of 300 nm at 1050°C flattened the surface to prevent liquid leakage after bonding, see figure S1B.


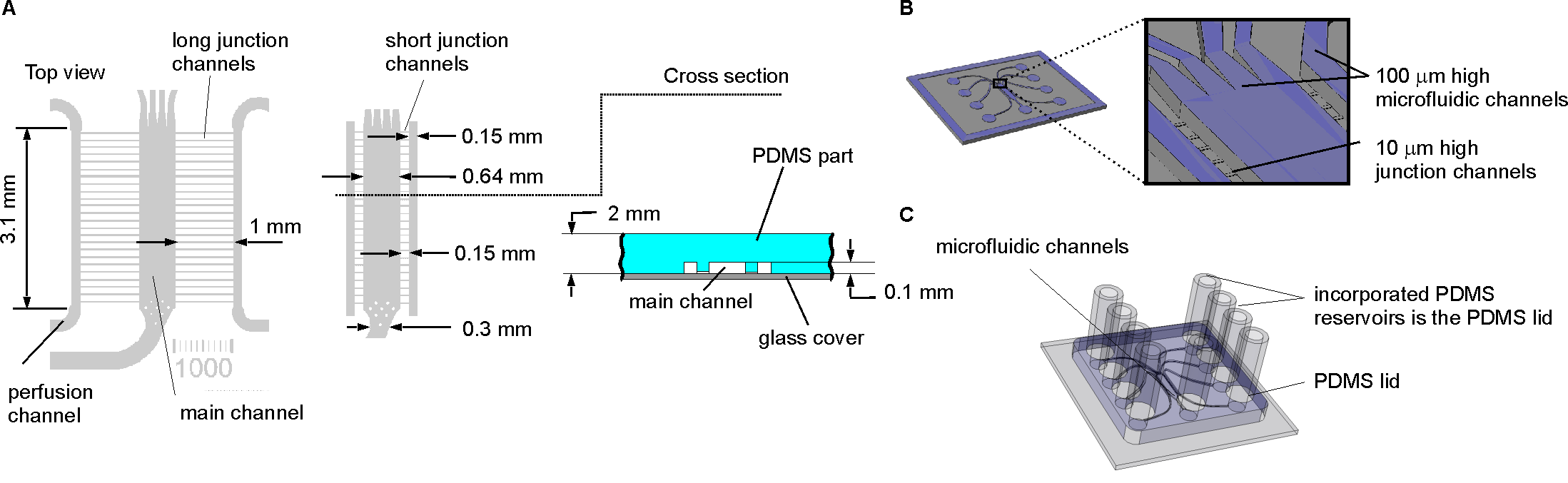


Figure S1, related to Figure 1: Microfluidic cell culture device design and fabrication: (A) Microchannel design and dimensions with long or short junction channels. Devices design comprises a culture channel, two perfusion channels and 24 junction channels. The culture and perfusion channels are 100 µm high, while junction channels are 10 µm high. Junction channel half pitches is 50 µm, with a width of 20 µm. (B) Silicon master for PDMS molding fabricated in the cleanroom with two-step lithography, deep reactive ion etching (DRIE) and wet surface oxidation. (C) PDMS cell culture devices with incorporated PDMS reservoirs. The microfluidic channels were sealed at the bottom by plasma bonding to glass.
