# Supplementary material for: Synergistic NGF/B27 Gradients Position Synapses Heterogeneously in 3D Micropatterned Neural Cultures
Source: PLoS One. 2011 Oct 13;6(10):e26187. doi: 10.1371/journal.pone.0026187 (PMC3192785; doi:10.1371/journal.pone.0026187)
Supplement: Supporting Information S2 — Chemical gradient characterization and modeling. Details on gradient measurements and modelling are presented. (DOC) [file pone.0026187.s002.doc]

*Chemical gradient characterization and modeling*

Gradient studies require maintenance of absolute concentration gradients ∇C, while providing the possibility to define average concentration C_avg_ and relative concentration gradients ∇C/C_avg_. Microfluidic systems are very suitable to control these concentration parameters. However, for neuronal cells ∇C has to be kept constant over weeks, because cells respond late to gradient environments. Stable gradient over long term can only be maintained through perfusion. Hence, large external reservoir systems and tubings have to be included into perfusion setups. On the one hand, tubes and reservoirs are exposed to air, which can cause microbacterial contaminations [1,2]. On the other hand, culturing embedded cells in a sterile and closed Petri-dish in incubators prevents contamination, but complicates perfusion. We developed a pipette based gradient generation method to perform gradient experiments with primary cortical neurons in the incubator over two weeks. The method consists of two steps (Fig. S2 A). First medium is added to one empty reservoir. Volume difference generates time dependent hydrostatic pressure and flow. Second, after 2h reservoir volumes are equal; the diffusion phase starts. Every second day volume in on reservoir is renewed restarting the first phase. An off bench experiment with fluoresceine characterizes stability and gradient profile achieved through our method (Fig. S2 B, C). Hydrogel diffusion coefficient was adapted in a finite element model (FEM) on extracted concentration profiles. Using FEM precise NGF-2.5S gradient profiles were calculated (Fig. S2 D, E) for the whole experimental period. NGF-2.5S gradient stabilizes after 2 days through the refilling procedure. During second phase, the concentration slope in the culture channels flattened about max. 2.5 %. Compared to constant perfusion averaged concentration values are 5.6 % lower during the refilling procedure. After 5 days relative gradient differences between perfusion and refilling reduced to 1.7 %. These gradient differences, between the refilling method and constant perfusion, and Step 1 and Step 2, can be neglected, as studies only provide duly neural cell responses for relative or absolute gradient differences above 10% [3,4].


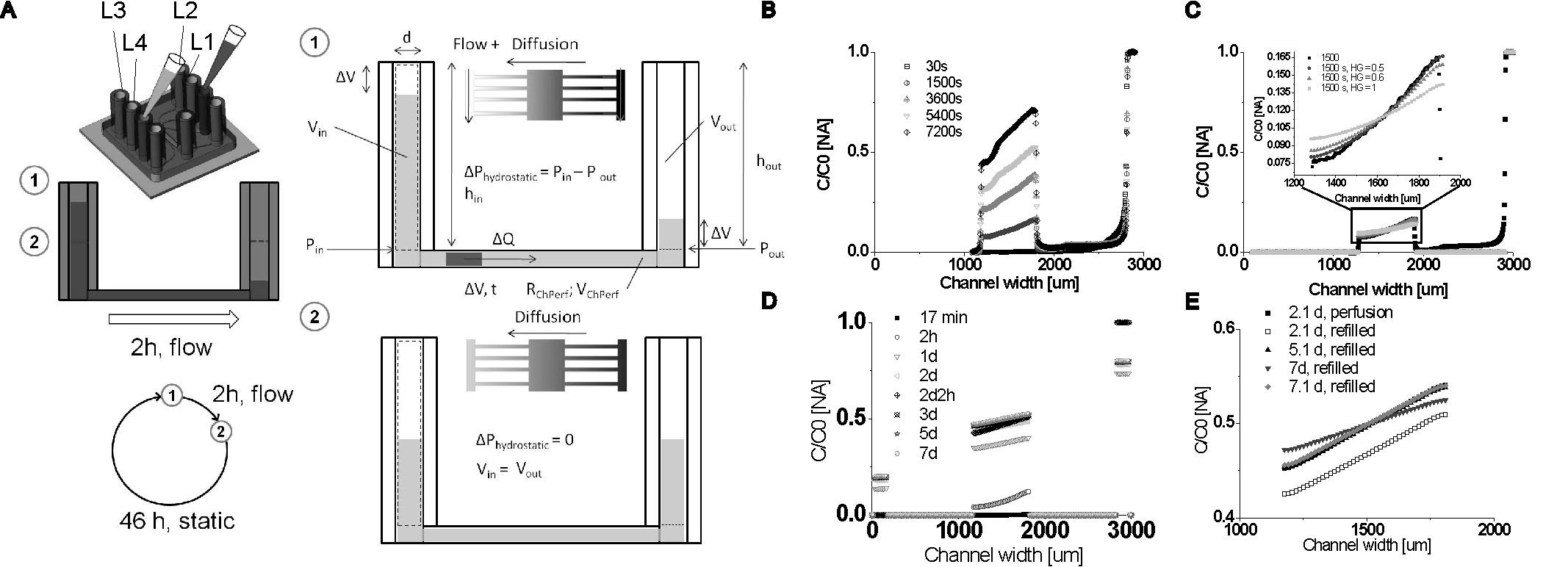


Figure S2, related to Figure 2: Gradient formation and finite element modeling (FEM) in the microfluidic device. (A) Stable gradients formed through (1) perfusion phase after reservoir filling and (2) diffusion phase. (B) Experimental gradient profile achieved with fluorescein. (C) Hydrogel diffusion coefficient adaption. (D) Stable formation of NGF gradients calculated with FEM.

**References:**

1. Wu M-H, Huang S-B, Cui Z, Cui Z, Lee G-B (2008) Development of perfusion-based micro 3-D cell culture platform and its application for high throughput drug testing. Sensors and Actuators B: Chemical 129: 231-240.

2. Ziolkowska K, Jedrych E, Kwapiszewski R, Lopacinska J, Skolimowski M, et al. (2010) PDMS/glass microfluidic cell culture system for cytotoxicity tests and cells passage. Sensors and Actuators B: Chemical 145: 533-542.

3. Dertinger SKW, Jiang X, Li Z, Murthy VN, Whitesides GM (2002) Gradients of substrate-bound laminin orient axonal specification of neurons. Proceedings of the National Academy of Sciences of the United States of America 99: 12542-12547.

4. Kapur TA, Shoichet MS (2004) Immobilized concentration gradients of nerve growth factor guide neurite outgrowth. Journal of Biomedical Materials Research Part A 68A: 235-243.
