# Supplementary material for: Synergistic NGF/B27 Gradients Position Synapses Heterogeneously in 3D Micropatterned Neural Cultures
Source: PLoS One. 2011 Oct 13;6(10):e26187. doi: 10.1371/journal.pone.0026187 (PMC3192785; doi:10.1371/journal.pone.0026187)
Supplement: Supporting Information S3 — Morphological evaluation through immunostaining. Chosen immunostainings are explained in detail and non specific binding issues in the hydrogel are discussed. (DOC) [file pone.0026187.s003.doc]

*Morphological evaluation through immunostaining*

Evaluating spatial synapse distribution and synaptic density are based on immunostaining. Pre-synaptic units were fluorescently labeled using synaptophysin. Figure S3 shows that local synapse distribution differs dependent on NGF and B27 concentration and that pre-synaptic units follow axonal staining, NF-L, but not dendrite staining, MAP-2.


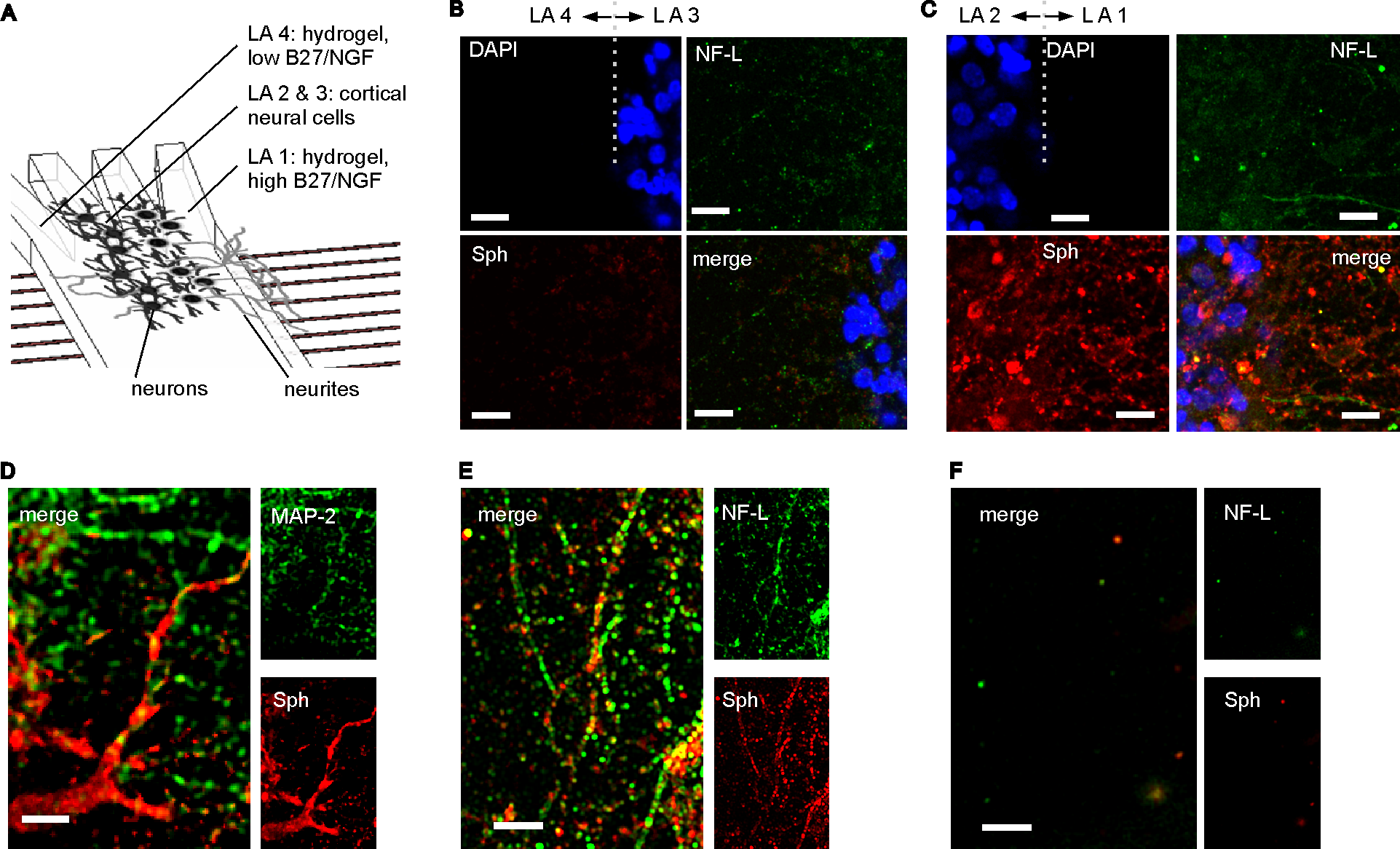


Figure S3, related to Figure 4: Local synaptic units are NGF/B27 concentration dependent. (A) Schematic of microfluidic device with local high (LA 4) and low (LA 1) B27/NGF concentration. (B, C) Different densities of synaptic units are formed at low or high NGF/B27 concentration. Immunostaining for axons with low neurofilament marker: NF-L (green), pre-synaptic marker: synaptophysin (Sph, red) and nucleus marker: DAPI (blue). (Bar = 10 µm). (D, E) dendrite (MAP-2, green) or axonal (NF-L, green) staining versus synapse staining (Sph, red, Bar = 10 µm). Contrast was enhanced for visibility. Pre-synaptic units follow axon neurofilament markers, but not dendrite markers. (F) Unspecific binding control of stained cell free hydrogel. Very sparse dots are related to unspecific bindings in the hydrogel.
