# Supplementary material for: Synergistic NGF/B27 Gradients Position Synapses Heterogeneously in 3D Micropatterned Neural Cultures
Source: PLoS One. 2011 Oct 13;6(10):e26187. doi: 10.1371/journal.pone.0026187 (PMC3192785; doi:10.1371/journal.pone.0026187)
Supplement: Supporting Information S4 — Evaluation of the cell response on the NGF/B27 gradient based on synapse formation. This file gives details how spatial synapse distribution was evaluated based on spatial fluorescence intensity measurements. (DOC) [file pone.0026187.s004.doc]

*Evaluation of the cell response on the NGF/B27 gradient based on synapse formation*

We studied different combinations of NGF and B27 gradients. To facilitate comparing spatial synapse distribution gradient trend curve was introduced. The trend curve is a linear regression fit on the fluorescence intensity measurements. Figure S4 shows the validity of a linear trend curve to discuss the output of different gradient effects based on synapse distribution.


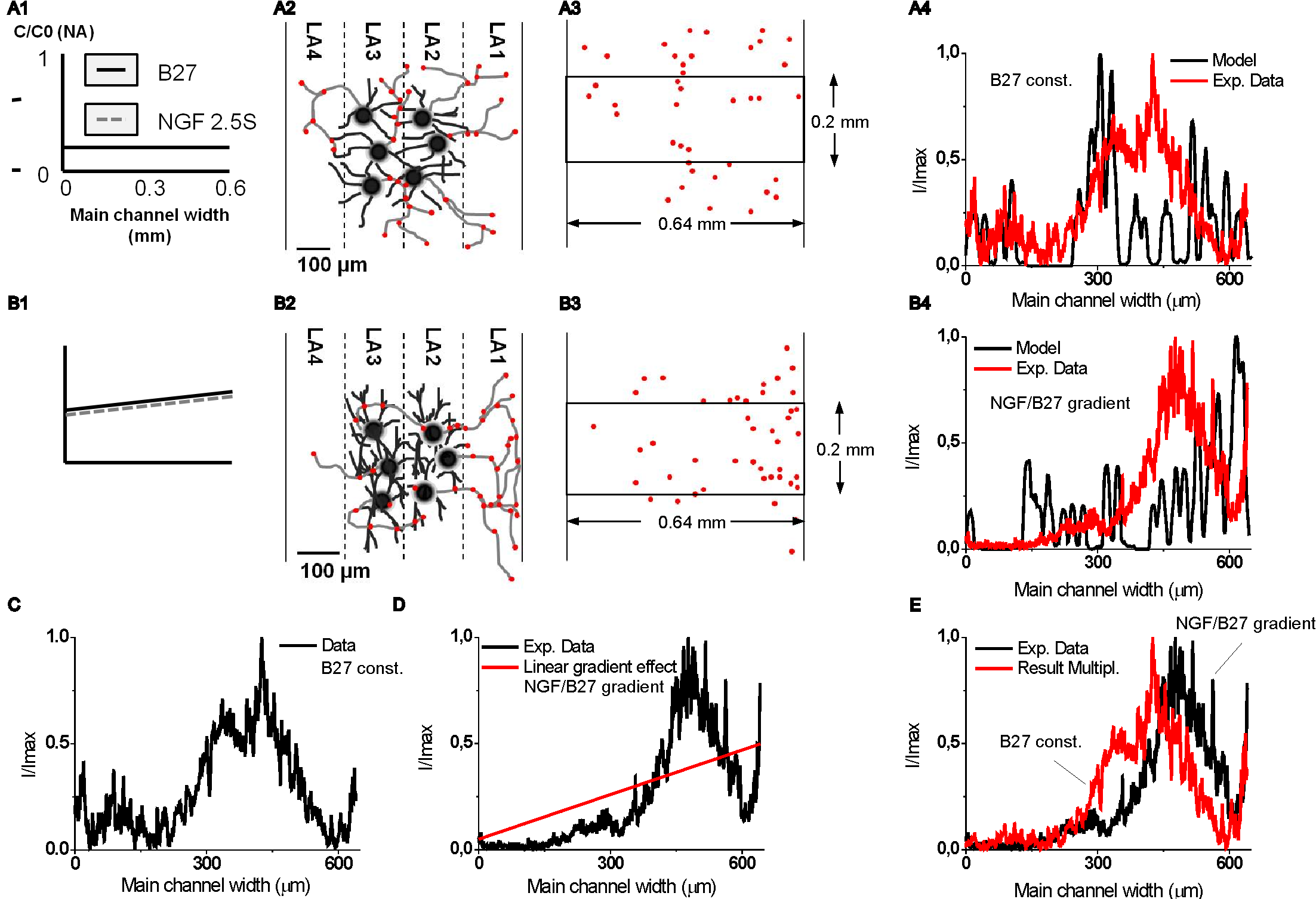


Figure S4, related to Figure 4 and 5: Generic model for spatial synapse formation in our micropatterned 3D neural cell culture. (A1, B1) Cell culture conditions of homogeneous B27 distribution versus NGF/B27 gradient. (A2, B2) Cartoon of general synapse formation established through B27 or NGF/B27 culture condition. Synapse to axon ratio is kept constant and synapses are homogeneously distributed over the axon length. Only axons have been randomly assembled and oriented. (A3, B3) Extracted synapse position from cartoons A2, B2 with region of interest for averaged surface intensity plots. (A4, B4) Normalized intensity plots over the main channel from generic cartoon compared to real experimental data. (C) Intensity plot of synapse distribution from neural cell culture with homogenous B27. (D) Synapse distribution under NGF/B27 gradient. Linear regression fit demonstrates gradient trend effect. (E) Data shown in (C) was multiplied with the gradient trend curve and normalized. The plot compares the multiplication result to the experimental data from NGF/B27 gradient. Both curves correlate, hence spatial synapse formation follows the induced gradient.
