# Supplementary material for: Synergistic NGF/B27 Gradients Position Synapses Heterogeneously in 3D Micropatterned Neural Cultures
Source: PLoS One. 2011 Oct 13;6(10):e26187. doi: 10.1371/journal.pone.0026187 (PMC3192785; doi:10.1371/journal.pone.0026187)
Supplement: Supporting Information S5 — Synapse distribution increases with a higher gradient slope. Additional results that show the increased spatial synapse distribution through increased gradient slope. (DOC) [file pone.0026187.s005.doc]

*Synapse distribution increases with a higher gradient slope*

In a side experiment we demonstrated that increasing the NGF/B27 gradient also increases the effect of spatial synapse distribution. Figure S5 introduces a microfluidic device parameter and how its dimensional change can influence gradient profile and synapse distribution.

**
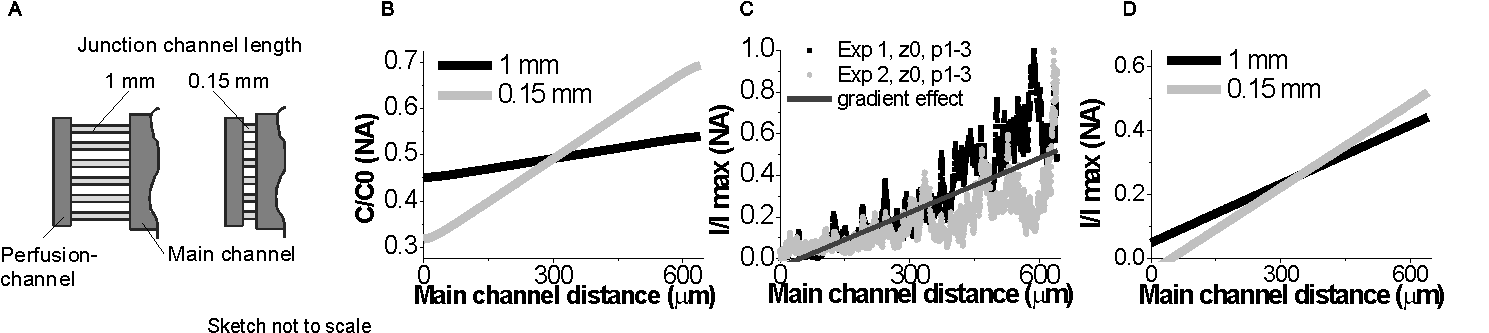
**

Figure S5, related to Figure 5: Synapse distribution scales with 12B27pNGF-53 gradient slope. (A) Two different lengths of junction channels. The length of the junction channels defines the gradient slope. The shorter the junction channel, the straighter is the chemical gradient slope. The disadvantage: during our refilling procedure the slope decreases faster with short junction channels. (B) Simulated concentration slopes for 1 mm and 0.15 mm junction channel length. (C) Synapse distribution in the main channel with 0.15 mm short junction channels. Linear gradient effect was fitted to consolidated plots. (D) Comparing synapse distribution in short (0.15 mm) and long (1mm) junction channel devices. The stronger the 12B27pNGF-53 gradient the stepper is the gradient effect.
