# Supplementary material for: Synergistic NGF/B27 Gradients Position Synapses Heterogeneously in 3D Micropatterned Neural Cultures
Source: PLoS One. 2011 Oct 13;6(10):e26187. doi: 10.1371/journal.pone.0026187 (PMC3192785; doi:10.1371/journal.pone.0026187)
Supplement: Supporting Information S6 — Evaluation of the cell response on the NGF/B27 gradient based on synaptic density. This file gives further details on evaluating spatial synapse distribution through determining local synaptic densities. (DOC) [file pone.0026187.s006.doc]

*Evaluation of the cell response on the NGF/B27 gradient based on synaptic density*

Spatial synapse distribution can also be evaluated by a quantification method that determines synaptic density. Figure S6 describes how we determined synaptic density. This method was used to extract native synaptic density in Fig. 1B from cortical layers taken from [5].


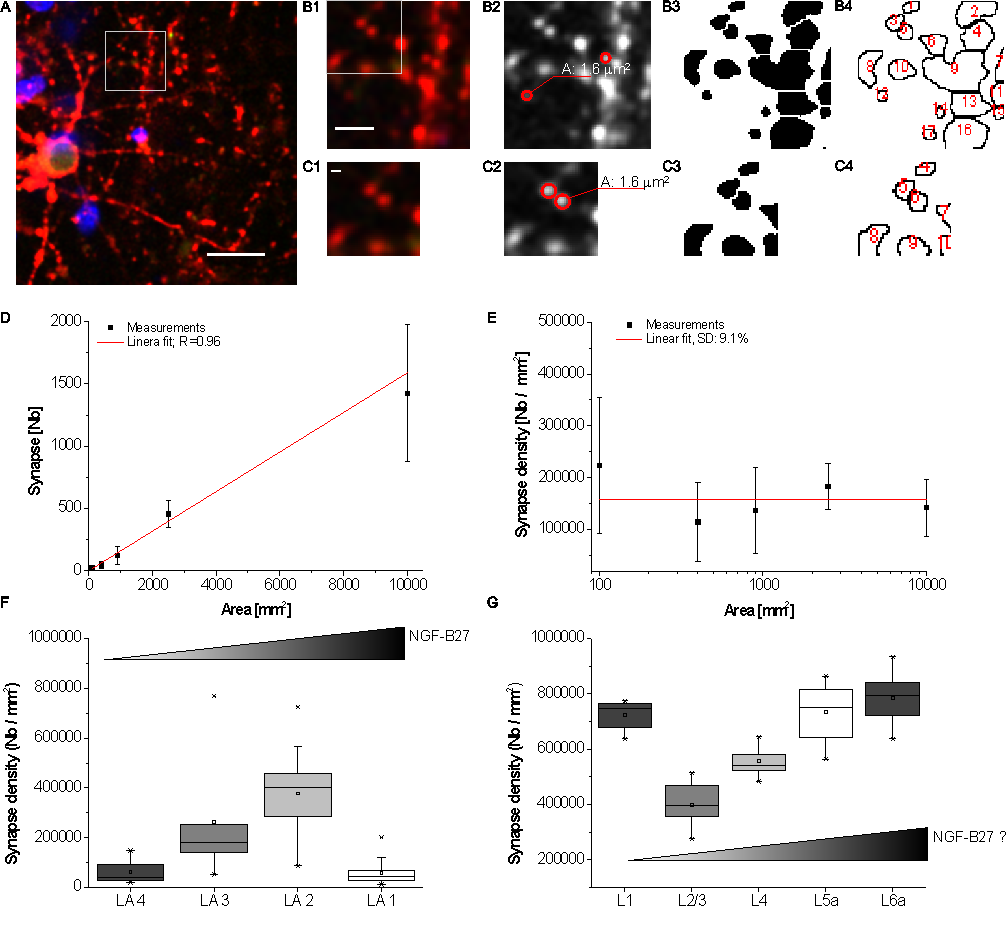


Figure S6, related to Figure 1, 6: Quantifying synaptic density. (A) Confocal fluorescence image, with cell nucleus (DAPI-blue) and pre-synaptic (synaptophysin-red) staining (Bar = 20 µm). (B and C, row) Image processing to determine surface area covered by synaptic units. (B1) Defined 30 x 30 µm^2^ area (Bar = 5 µm) or (C1) 10 x 10 µm^2^ (Bar = 1 µm) were extracted from original confocal images. (B2, C2) Red channel image was converted into 8-bit gray scale image. In our image data smallest synapse cycles covered an area of 1.6 µm^2^. (B3, C3) An image threshold is set to subtract unspecific binding. (B4, C4) Total synaptic surface is counted using particle detection. Numbers of synapses were extracted by dividing total synaptic surface through single synaptic surface. (D) Automatic counting of synapses correlates linear with the increase of surface area of interest. (E) Surface normalized number of synapses shows no significant different synaptic density between different surface areas (one-way ANOVA, p < 0.01). Linear fit gave 9.1 % standard deviation. (F) Layer dependent synaptic densities were extracted from micropatterned neural cell cultures under NGF/B27 gradient condition in 50 x 50 µm^2^, n = 16. Cells are positioned in LA 3 and LA 2(G) Native, layer dependent synaptic density, extracted from cortical layer images in literature [5]. High resolution images from array tomography made synapse extraction in 10 x 10 µm^2^ possible, n = 16 for each layer.

**References:**

5. Micheva KD, Busse B, Weiler NC, O'Rourke N, Smith SJ (2010) Single-Synapse Analysis of a Diverse Synapse Population: Proteomic Imaging Methods and Markers. Neuron 68: 639-653.
